# Type and Location of Wearable Sensors for Monitoring Falls during Static and Dynamic Tasks in Healthy Elderly: A Review

**DOI:** 10.3390/s18051613

**Published:** 2018-05-18

**Authors:** Rosaria Rucco, Antonietta Sorriso, Marianna Liparoti, Giampaolo Ferraioli, Pierpaolo Sorrentino, Michele Ambrosanio, Fabio Baselice

**Affiliations:** 1 Department of Motor Sciences and Wellness, University of Naples “Parthenope”, 80133 Naples, Italy; marianna.liparoti@uniparthenope.it; 2 IDC Hermitage Capodimonte, 80133 Naples, Italy; pierpaolo.sorrentino@uniparthenope.it; 3 Department of Engineering, University of Naples “Parthenope”, 80133 Naples, Italy; antonietta.sorriso@uniparthenope.it (A.S.); michele.ambrosanio@uniparthenope.it (M.A.); fabio.baselice@uniparthenope.it (F.B.); 4 Department of Science and Technologies, University of Naples “Parthenope”, 80133 Naples, Italy; giampaolo.ferraioli@uniparthenope.it

**Keywords:** falls in healthy elderly, fall risk assessment, fall prevention, fall detection, wearable sensors

## Abstract

In recent years, the meaning of successful living has moved from extending lifetime to improving the quality of aging, mainly in terms of high cognitive and physical functioning together with avoiding diseases. In healthy elderly, falls represent an alarming accident both in terms of number of events and the consequent decrease in the quality of life. Stability control is a key approach for studying the genesis of falls, for detecting the event and trying to develop methodologies to prevent it. Wearable sensors have proved to be very useful in monitoring and analyzing the stability of subjects. Within this manuscript, a review of the approaches proposed in the literature for fall risk assessment, fall prevention and fall detection in healthy elderly is provided. The review has been carried out by using the most adopted publication databases and by defining a search strategy based on keywords and boolean algebra constructs. The analysis aims at evaluating the state of the art of such kind of monitoring, both in terms of most adopted sensor technologies and of their location on the human body. The review has been extended to both dynamic and static analyses. In order to provide a useful tool for researchers involved in this field, the manuscript also focuses on the tests conducted in the analyzed studies, mainly in terms of characteristics of the population involved and of the tasks used. Finally, the main trends related to sensor typology, sensor location and tasks have been identified.

## 1. Introduction

Falls and related accidents are a common and serious problem not only in a pathologic condition like Parkinson’s Disease [1], stroke [2] or multiple sclerosis [3], in which they are due to the motor and cognitive characteristics of the specific disease, but also for healthy people aged 65 and over [4]. A fall is defined as coming to rest on the ground or floor or other lower level, suddenly and involuntarily [5,6].

Many epidemiological studies have reported a fall frequency of 28–35% in adults aged 65 and over [7,8,9], highlighting the need of developing effective and inexpensive ways to predict and prevent risk factors. [5,6,10].

Although some falls are probably unavoidable, most of them are due to the combination of intrinsic and extrinsic risk factors [11]. Intrinsic risk factors are related to the subject’s characteristics, which include immutable biological features, sedentary lifestyle, concomitant presence of pathologies and use of medicines, age-related changes such as cognitive impairment, gait pattern alterations, and inability to maintain postural stability [12]. Musculoskeletal weakness [13] and decline of cognitive functions are known to be correlated with falls risk [14]. More specifically, a close relationship between motor and cognitive functions in both healthy elders and cognition-compromised subjects has been observed [15,16].

Nevertheless, falls may also depend upon extrinsic factors, including environmental, ergonomic and organizational aspects (inadequate housing, insufficient illumination, hazards in domestic and public places, lack of social and health care services and restricted social interaction) [12].

As reported in [17], in most cases, falls occur as the result of the individual’s inability to adapt to environmental conditions. For example, increasing the slipperiness of a floor surface (i.e., from dry to wet) creates a high risk of slipping. In fact, the forces of the foot that are normally generated during gait require friction to counteract the shear forces and maintain balance. When the friction available at the level of the shoe can not meet the biomechanical requirements, a slip becomes more likely, and the consequent risk of injury with it.

In the case of highly predisposing risk factors, the frequency of falls increases, leading to a growth in the mortality rate and in the probability of causing/worsening disabilities [18]. Fractures of the femur, head injuries, damages to the lower and upper limbs and post-fall syndrome are fall-related events, which could cause a loss of confidence, hesitation and, consequently, a reduction of the quality of life [4,6,19]. The incidence of fall-related mortality is greater in men [6] than in women, although the latter show a higher prevalence of falls. The incidence of mortality is higher in men probably due to more comorbidities or to the tendency to not seek medical care until the condition becomes severe, or to delay to the access to prevention and management of diseases [6]. Furthermore, men are usually involved in intense and dangerous physical activity and risky behaviours that predispose to major risk of fall. Many epidemiological studies predict twice as many injuries up to the year 2030 [6,20].

Fall-related costs can be categorized in direct costs (which include medical and diagnostic examinations, rehabilitation treatments) and indirect costs (which have an impact on family economy) [4]. Some injuries related to falls, in addition to physical and psychological consequences on the subject, require a rehabilitative or surgical treatment, resulting in an increased hospitalization time, which has a significant economic impact on the healthcare system [6,21]. Indeed, this results in the highest direct costs, which represent about 50% of the total share in the case of falls, as shown by epidemiological data from the World Health Organization (WHO) [6].

Given the high incidence of falls in healthy elderly, in order to prevent them, it is necessary to identify predisposing risk factors, to analyze subject specific needs and to use a targeted preventive strategy, like behavioral changes [6] or promoting a healthy lifestyle [22], despite some evidence showing the opposite [23,24]. In addition, in order to make elderly people less predisposed to falls, it would be useful to plan age-friendly environments through the installation of staircases, adequate lighting and non-slip rugs [25].

The interest in the studying of mechanisms of balance and body-orientation control has increased in the last years since it has proved to be an effective tool for fall monitoring. Different techniques of measurement and assessment have been used to quantify the postural stability in elderly individuals both in static and dynamic conditions [26,27].

Human posture can be defined as “the position of one or many body segments in relation to one another and their orientation in space” [28]. Head, trunk, pelvis, lower limbs and feet are known as body “segments”, whereas spinal, hips, knees, ankle and shoulder joints are denoted as body “linkages” [29]. Human posture depends on a great number of factors—among them, we cite the muscle tone [30], the orientation and the equilibrium [31], which are involved in the control of the body position with respect to the walking surface (the relationship between the Center of Mass (CoM) and Base of Support (BoS) [32]), and in the control of alignment of Center of Pressure (CoP) with respect to the Center of Gravity (CoG) [26].

According to a biomechanical approach, the falls could be due to a change in position of the CoM projection beyond the BoS without any correction [10]. Even when standing, the body sways constantly and for this reason such posture is called static standing or stand still. The most common way to study the balance in static standing is through the trajectories of the CoP with respect to the CoG [33,34,35], although nonlinear measures have been proposed in some studies [36,37,38]. The assessment of the degree of postural sway during static standing could be useful to evaluate the ability to preserve balance control. For this reason, participants are commonly asked to perform a static upright task, on a force platform, in open- and closed-eyes condition, with both arms along the body [39,40]. During this test, it is also possible to analyze the relationship between the CoM and CoP, it being useful for fall prediction [39] and risk assessment [40].

One of the major health issues for an older adult is the loss of stability or a slip and the subsequent fall during walking. For this reason, the evaluation of dynamic stability, the assessment of activity and mobility in daily living conditions (for example, through ADLs) or through tasks such as simple walk, “time up and go” (TUG) test [41] and the 6-minute walk test [42], could provide useful motor outcomes to detect the early signs of balance alteration and then provide key information about fall risk [43]. The latter results could, however, be capitalized in a predictive fashion so as to accomplish fall detection/prediction. Many articles in the literature are focused on defining gait characteristics in the elderly with the aim of identifying the causes of fall. In the recent review article by [27], the authors tried to evaluate the sensitivity of biomechanical measures that quantify gait stability in older populations. In detail, they analyzed different approaches to study gait stability based on two different classes: (i) linear variability of temporal measures (such as swing and stance time, step width, stride velocity), and (ii) (nonlinear) orbital or local stability measures (such as Floquet multipliers or Lyapunov exponents). They concluded that, although some biomechanical approaches for determining specific parameters of mobility can assess the function and stability in the elderly, they hardly have been taken up in clinical settings because of their unclear sensitivity and specificity, together with the time and effort required for their use. Within this framework, a key role is played by the adopted sensing technologies, both in terms of hardware and software approaches.

Recently, the interest in falls is increased not only in healthy subjects, but also in pathological conditions such as Parkinson’s disease [1,44,45], multiple sclerosis [3,46], stroke [2,47] and Alzheimer’s disease [48,49]. However, this review investigates the studies published in the last two decades in the field of stability control for fall risk assessment, prevention and detection based on wearable sensors in healthy elderly people only. This is because, in a pathologic condition, the stability, and therefore the fall risk, strongly depends on the motor and cognitive characteristics of the disease.

An interesting review can be found in [50], where the authors focus their attention on wearable inertial sensors for fall risk assessment in geriatric populations. Within this manuscript, we extend the analysis to other sensor typologies, such as pressure sensors, electromyographic sensors and cameras, and to fall detection and prevention. Therefore, the aim of our work is to provide an overview of the most adopted sensing technologies in these fields, with a focus on the type of sensors (rather than algorithms), their position on the body and the kind of tasks they are used in. The existing literature has been found by defining a search criteria and considering some of the most important publication databases. In more detail, the search criterion is reported in Section 2, and the search results are reported in Section 3. Starting from these outcomes, some trends are identified and reported in Section 4. Finally, conclusions are drawn in Section 5.

## 2. Methods

This section is focused on the definition of the research criteria and strategy. After the introduction of the keywords, which are required in order to correctly understand the flow chart of the selection procedure, the framework of the research strategy is proposed and motivated. Finally, an overview of the results is briefly summarized in the last subsection.

### 2.1. Keywords Definition

In this section, a brief description of the main keywords is provided in order to clarify the search criteria.

*"Aged"*: with this term, we considered all those works whose studies either take into account the stability analysis for subjects aged over 64 years or consider a younger population for a preliminary assessment but with an intended application to old people.

**Figure 1:**
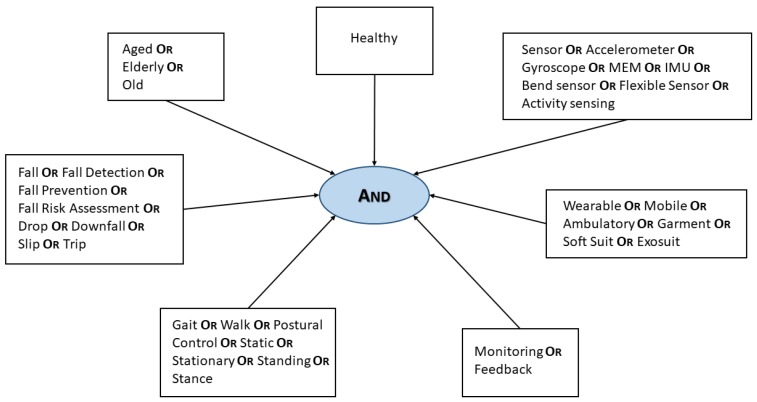
Block scheme of the adopted search strategy for the papers selection.

*“Postural control”*: in biomechanics, it refers to the ability of maintaining the line of gravity (vertical line from centre of mass) of a body within the base of support with minimal postural sway. In order to classify the different articles, we adopted the terms reported in Figure 1 both for the *dynamic analysis* (i.e., “gait” and “walk” words) as well as the *statical analysis* (i.e., “static”, “stationary”, “standing” and “stance” words). The former refers to the study of a walking subject, while the latter investigates the function of the balance system during quiet stance.

*“Fall detection”*: it refers to the drop from a standing position or during an activity. Falls are a major public health problem among old people and the use of a fall detection system as an assistive device is important to alert when an event of fall occurs.

*“Fall prevention”*: it refers to all those articles that analyze strategies aimed at preventing the fall of older people, including doing exercises to improve muscle strength and balance, and making simple changes to home or wearing sensible shoes.

*“Fall risk assessment”*: it refers to those manuscripts that focus on the cause of falls, such as environmental issues, age, mental state and mobility.

*“Wearable sensor systems”*: national and international guidelines on fall prevention all reinforce the need to screen and assess older people, also with an attempt to identify the ones with increased risk of falling. In order to achieve this goal, one of the adopted solutions is to use wearable or portable sensors systems.

### 2.2. Literature Research Strategy

The aim of the manuscript is to provide an overview of the type and location of wearable sensors for the monitoring and assessment of falls during static and dynamic tasks (i.e., walking and standing) in healthy elderly people [51,52,53]. For this purpose, a bibliographical research on the most important scientific publication databases was performed. In particular, an approach similar to the one proposed in [54] in case of the newborns has been followed. We focused on IEEE Xplore, SpringerLink, Science Direct and PubMed databases. Articles related to both dynamic and static stability analysis have been taken into account, since, as previously reported, there is a close relationship between falls and static (i.e., [26,55,56,57,58]) or dynamic (i.e., [27,59,60,61,62]) tasks. In order to perform this research, the set of keywords previously introduced has been applied. Figure 1 illustrates the flow chart of the selection process. The key point is represented by the AND block, which merges the other peripheral branches, in which each word is related to its synonyms by means of an OR condition. By doing so, the research is carried out via the intersection of at least one word per each peripheral block, otherwise the result of the query is null.

### 2.3. Study Selection and Screening Process

**Figure 2:**
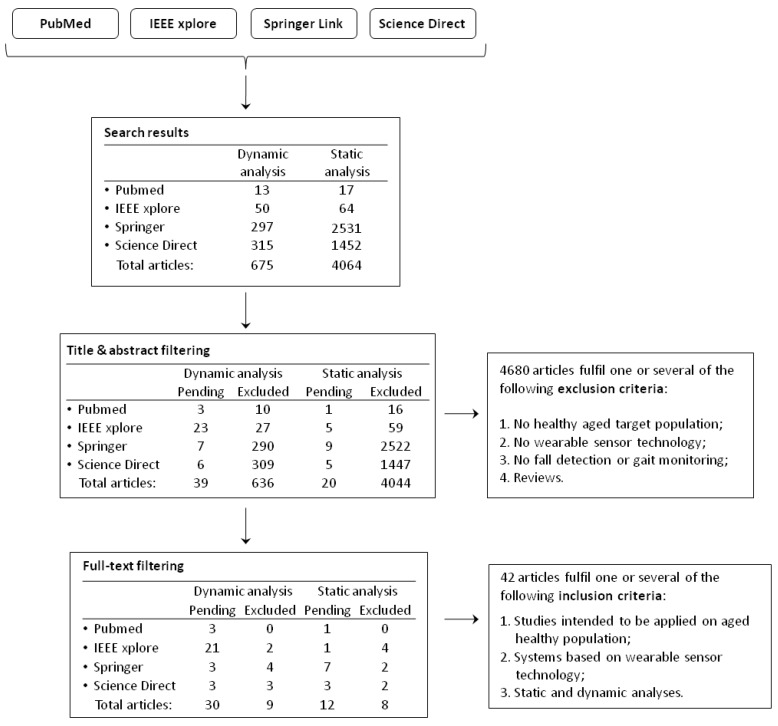
Adopted research methodology. The flow chart illustrates the two steps of the selection procedure (title and abstract filtering and full-text reading).

After a preliminary search, 675 results have been found for the dynamic task and 4064 articles for the static task, as shown in Figure 2. In the first step, based on paper titles and abstracts, the following exclusion criteria have been identified:

- all the studies that do not include healthy aged population as target;
- articles not including a wearable sensor technology;
- all the manuscripts not related to “fall risk assessment”, “fall prevention”, “fall detection” and “standing analysis”;
- all the review articles.

This selection drove into the exclusion of 4680 articles. Regarding the remaining 59 manuscripts, a second refinement step has been performed, which consists of a full-text reading of the articles. This filtering process was based on the following inclusion criteria:

- all studies on healthy aged people or intended to be applied on an aged population;
- all systems based on a wearable sensor technology;
- gait and standing analysis have been included.

Finally, 42 articles remained and were considered in this work. In more detail, we started the selection procedure by considering all the previous databases for collecting the articles, in order to provide more information regarding their distribution among the databases, although most of the papers were present on Pubmed. Therefore, we decided to indicate with the “Pubmed” label all those articles not present on the other databases.

**Figure 3:**
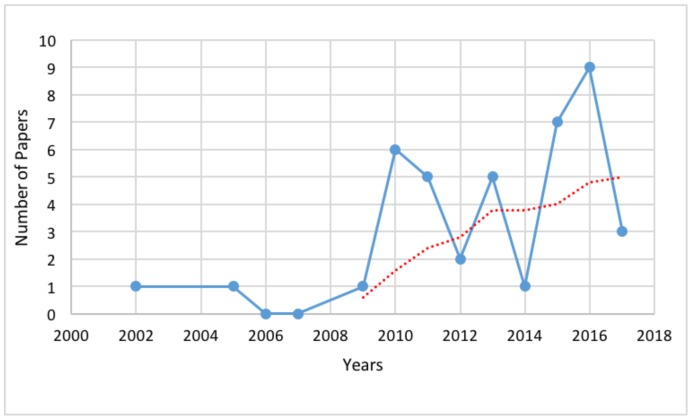
Number of found manuscripts that have been published in the last 15 years (blue continuous line) and its average (red dotted line).

Figure 2 provides an overview of the whole selection process and the results of the literature research are reported in Table 1. In addition in Table 2 are reported the acronyms for the tasks, in Table 3 the acronyms for the sensor positions while in Table 4 the acronyms for the sensors types, used in Table 1. Starting from these results, the quantity of works published in last years, both in conference proceedings and journals, the number and age distribution of participants, the number and typology of sensors and the types of conducted tasks have been reported as graphs. In Figure 3, the distribution of the manuscripts of Table 1 is shown. In order to make the trend more clear, a five point moving average has been applied, resulting in the red dotted line of Figure 3. It is evident that the topics of fall risk assessment, falls monitoring and falls preventing in elderly people have gained increasing interest in last years, with an almost linear growth of the published manuscripts.

The manuscripts resulting from the selection have been critically analyzed in order to provide an overview of the general trends and of the most adopted technologies in the field of falls detection, falls risk assessment and falls prevention in healthy elderly. The analysis has been carried out according to three aspects: the characteristics of the participants involved in each study, the approaches for sensing and the typology of tasks that the cohort has been asked to perform.

## 3. Results of the Bibliographic Research

The papers resulting from the selection are summarized in Table 1 in a compact way. We chose to categorize the papers according to three main aspects: the participants involved in the study, the adopted sensors and the body portion under focus. The assessment of the participants gives an idea of the target of the study in terms of subjects, and defines if one is dealing mainly with a technical experiment of with clinical research. The second aspect allows to identify the technical features of the research and its costs. The analysis of the body parts mainly involved in the study allows to define the main biomechanical aspects considered in the work. Relevant information about such parameters can be found in the columns of the table. In more details, papers have been organized according to the following aspects:

- *Author (year)*: the family name of the first author is reported, and the publication year between brackets;
- *Participants (number/age)*: the number of volunteers considered in the study. For each population, the number of the subjects involved and their average age is reported;
- *Number of sensors*: how many sensors are used (simultaneously or not) within the study. In the case of compound sensors, each of the elementary sensing technology has been accounted for;
- *Sensor type*: the typology of sensors adopted in the study. Their acronyms are reported in Table 4. In case smartphones have been employed, we only reported the name of the actual sensors;
- *Sensor placement*: sensor attachment location;
- *Task*: the (one or more) task the participants have been asked to perform for the validation of the study. We chose to include activities implying oscillations of the center of mass, as they make falls more likely;
- *Analysis*: the type of performed analysis, i.e., static, dynamic or both.

## 4. Discussion

### 4.1. Participants

**Figure 4:**
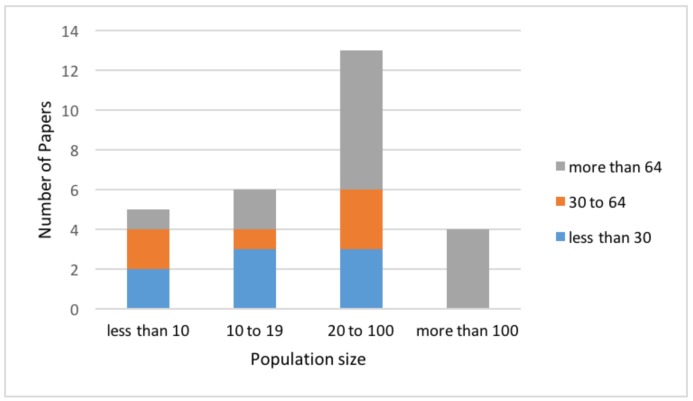
Participant age distribution in the case of different population sizes.

In Figure 4, we report the number of participants for each study (grouped in four categories) for the considered articles, excluding the ones that do not specify the population age and/or participants’ number, and that discuss a specific sensor/methodology only qualitatively, without any population involved. From the inspection of the figure, the most frequent bins are very different from a numerical viewpoint. Additionally, only a few articles have more than 100 participants in the study, reflecting the difficulty in recruiting a wide audience for these studies.

From the conducted review of the literature, it emerges that there are mainly two lines of research: (a) studies concerning the comparison between old versus young subjects [42,65,71,84] and (b) works aiming at underlining some peculiar differences between groups of elderly fallers and non-fallers [43,79,80,81]. In both cases, there is a prevalent presence of healthy elderly groups. This observation is confirmed by Figure 4, where it is shown that most of the participants in all studies are 64 years old or more, which is not surprising provided that this was used as an inclusion criterion. On the other hand, the prevalence of the young and middle (30 to 64) groups in studies with less than 10 subjects reflects the need of a fast validation in the case of a novel instrumental technology.

### 4.2. Typology of Sensors

By considering the sensors adopted in the studies, a first important consideration can be drawn. Specifically, most of the proposed sensing methodologies are simple from a technological point of view, as a significant share of the works adopt a few sensor technologies for monitoring the subjects: 16 papers use only one sensor and 20 papers use two sensors. On the other hand, only a small percentage of the papers proposes the joint use of three or more sensors types for improving the monitoring performance. Given the above numerical categorization (e.g. single sensor, two sensors and ≥3 sensors, respectively), we proceed in what follows with category-specific observations.

#### 4.2.1. Single Sensor

**Figure 5:**
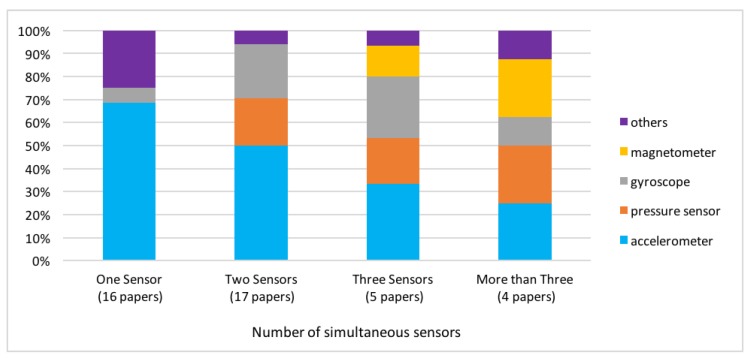
Sensor typologies in the case of papers exploiting one, two, three or more than three sensors.

From Figure 5, it can be seen that the accelerometer is by far the most adopted sensor in the group of single-sensor type paper with a percentage of adoption of about 70%. This is probably due to the low-cost and to the plethora of devices available on the market, most of them with integrated wireless communication system, small size, weight and long-lasting batteries. For example, Cola et al. [68] propose a method requiring the use of a single waist-mounted accelerometer to achieve a continuous monitoring of deviation in the gait of elderly people. Among the single sensor approaches, about one fourth of the papers adopts a wide range of sensor technologies, both commercial products (e.g. Kinect, Wii, cameras) and prototypes. For instance, Diraco et al. [73] propose a non-invasive technique for posture classification suitable to be used in several in-home scenarios. This procedure exploits 3D point cloud sequences acquired by using a single time-of-flight sensor to classify the posture hierarchy by using a support vector machine (SVM) approach. Conversely, from the cameras proposed in [73], Najavi et al. [86] propose a commercial prototype. A more recent technology can also be employed for gait analysis and fall detection. Stone et al. [91] monitored a population of aged people (67–97 years old) for a few months using a Microsoft Kinect sensor (Redmond, WA, USA) in order to evaluate fall risk by means of the standard methodologies timed-up-and-go (TUG) time and habitual gait speed (HGS) tests.

#### 4.2.2. Two Sensors

By focusing on the papers that use two sensors, two families can be identified: approaches that combine the accelerometer with a pressure sensor (generally placed within the shoes), and methodologies that jointly use the accelerometer and the gyroscope sensors, usually placed on the same electronic board. For instance, the study proposed in [75] introduces a new robust classifier for sit-to-stand (SiSt) and stand-to-sit (StSi) detection in daily activity based on both gyroscope and accelerometer. The monitoring system consists of a single inertial sensor placed on the trunk. By using dynamic time warping, the trunk acceleration patterns of SiSt and StSi are classified based on their similarity to specific templates. Gopalai et al. [76] also use accelerometers and gyroscopes to improve postural control and shorten rehabilitation periods among the young and the elderly. Beyond such “standard” sensors, the use of some peculiar devices can be explored for the stability control of elderly people. Greene et al. [77] investigate the gait variable Minimum Ground Clearance (MGC) using shank-mounted inertial sensors containing both a tri-axial accelerometer and an add-on tri-axial gyroscope. The aim is to estimate clinically meaningful parameters, which may be used in the screening for falls risk.

#### 4.2.3. Three or More Sensors

In the case of three or more devices, other sensing technologies are adopted, including magnetometer, camera or electromyography (EMG), as described in [64], where Bertolotti et al. have designed and built an autonomous wearable 9-degrees-of-freedom system embedding three axial accelerometers, three-axial gyroscopes, and three-axial magnetometers. In [88], a support system for detecting falls of an elder person is presented. This system is an AAL (Ambient Assisted Living) system that allows to infer a potential fall by the combination of a wearable wireless sensor node, based on an accelerometer, and a static wireless non-intrusive sensory device, based on heterogeneous sensor nodes. The sensors network would spread throughout the environment, in any room of the house, routing and linking nodes to the base station. The wearable node is not intended for determining a falling situation, but to advise the reasoner layer about specific acceleration patterns that could eventually imply a fall.

### 4.3. Position of Sensors

**Figure 6:**
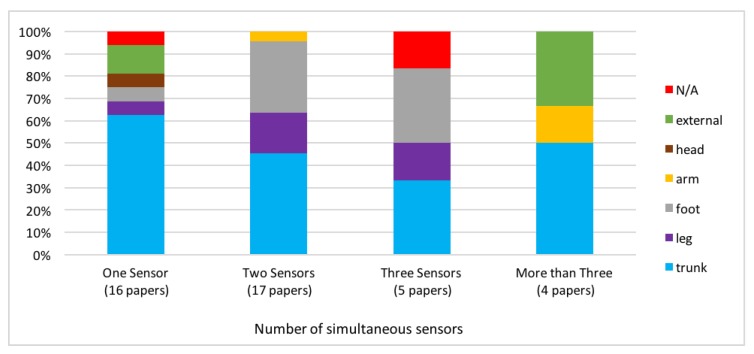
Sensor locations in case of papers exploiting one, two, three or more than three sensors.

Regarding the position of the sensors, 25 studies monitored two or three points of the body (Figure 6). Most importantly, the placement of sensors on the human body is mostly on the trunk.

Among all, the work of Curone et al. [70] needs to be mentioned, who developed a processing methodology to monitor the posture and detect the activity level of the subject based on data from an accelerometer. The sensor is placed on the upper trunk, inside a garment similar to a jacket. Moreover, the algorithm is able to associate a reliability value in order to launch alarms only in case of effectively dangerous conditions. The methodology exhibits a very high accuracy in task classification (about 96%), although the evaluation has been done on a very small cohort (6 participants). In [86], authors present a method for evaluating postural transitions. The algorithm analyzes the parameters in the wavelet domain, and related them to the falling risk of the subject. In addition, Ref. [68] monitors the trunk for its analysis, reaching a very interesting accuracy value (84%) in the case of a 30 people cohort. Unfortunately, no evaluation is done in the case of older subjects.

Besides the trunk, sensors are usually placed on the foot (almost 30% of all selected studies, corresponding to pressure sensors within the shoes) and the leg, when one to three sensors are considered. In addition, the inertial device presented by Bertolotti [64] provides objective measurements of limb movements for the assessment of motor and balance control. It can be adopted for the fall risk assessment, quantifying sports exercise, studying people habits, and monitoring the elderly. Several acquisition configurations are presented as the sensors can be positioned on the trunk, on the thighs, on the arms and also on the head. In [75], the authors adopt inertial sensors positioned on the trunk and on the leg to monitor subjects, reaching high accuracies in classifying postural transitions (up to 95%).

Very few works adopt not-body worn sensors for monitoring subjects, although such kind of systems have the advantage of being totally non invasive. In particular, in [69], a video system is proposed for monitoring Alzheimer’s patients. A software module has been developed in order to recognize both physical tasks and instrumental activities of daily living. The proposed video processing algorithm is quite effective in the physical task recognition, achieving an F- score of 93. The approach proposed in [73] uses time of flight sensors for collecting 3D point cloud sequences and classifying the subject’s activities. Such methodology is interesting and capable of good performances, at least without any partial occlusion of the subject.

The reason why the trunk is the most used segment for the sensor location is tightly coupled to the upright gait, being a human characteristic that requires the ability to preserve the upper body balance during walking. Interestingly, in 1992, Perry [100] defined gait analysis as centered on the lower limbs, in particular on hip, knee and ankle joint kinematics, while defining the upper body as a mere “static passenger unit” of the locomotor apparatus. Later, empirical evidence greatly changed this point of view. Indeed, in recent years, many studies have moved their focus and confirmed that the trunk plays a fundamental role both in static and in dynamic stability, see for example the study of Bertolotti et al. [64]. Furthermore, in [68], the authors used a single waist-mounted accelerometer in order to assess frailty and risk of falls in the elderly. Curone [70] designed an algorithm that analyzes, in real time, the signals produced by one three-axial accelerometer placed on the trunk, in order to classify human activities and posture transitions. In addition, Karel et al. [84] used a a triaxial accelerometer worn at the level of the sacrum to evaluate the incidence of stumbling in the elderly in daily life.

A loss of gait stability in older people is documented in many studies and the corresponding literature shows that different aging factors, such as the loss of muscle strength [101], the decline in vision and the peripheral and vestibular sensations [102], directly hamper the ability to keep the upper-body stable during walking. According to these results, the scientific interest in the trunk increased in recent years, and the development of wearable sensor technologies, which enable reliable measurements of trunk movements, has contributed to such increased interest.

As observed earlier, there is a significant adoption of pressure sensors within the shoes. The reason is that monitoring the CoP both in static and dynamic conditions, together with measuring spatio-temporal gait variability, can identify the fall risk, and help reducing it. In particular, Aminina [42] proposed a 3D method for gait analysis using the foot orientation and trajectory during each cycle based on two inertial modules worn on each foot. Using data transmitted by the modules during long distance walking periods, a dedicated algorithm provides relevant gait parameters for the evaluation of the outcome of the rehabilitation program for fall prevention in elderly subjects. In addition, Di Rosa [72] used a pair of electronic insoles, for the “Wireless Insole for Independent and Safe Elderly Living” project, in order to assess the risk of falls through a novel Fall Risk Index based on multiple gait parameters and gait pattern recognition.

### 4.4. Tasks

**Figure 7:**
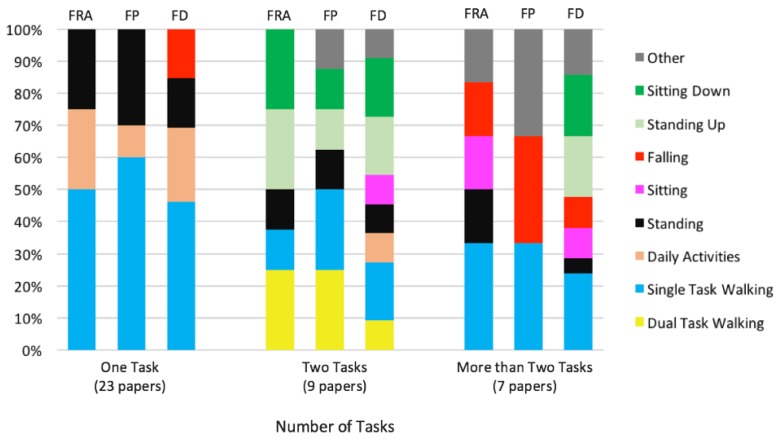
The type of tasks the participants have been asked to perform is case of one, two or more activities for fall risk assessment (FRA), fall prevention (FP) and fall detection (FD). Notice that (a) three out of all the articles listed in Table 1 were not reported in the figure (because they did not perform any test) and (b) to avoid an excessive number of categories, less investigated activities were clustered within “other” label.

The assessment of the ability to maintain the balance in static and dynamic conditions and the estimation of the risk factors of falls in older people has been done by asking the participants to perform one or more tasks, whether simple or complex. In Figure 7, the distribution of the different types of task in case of one, two or more required activities is reported. We have divided the “Task” paragraph accordingly.

#### 4.4.1. One Task

Among the papers selected in this study, roughly 23 works include in the experimental protocol the execution of one task only. As it can be see in Table 1, 15 papers focus on the analysis of dynamics using one task, such as a walk at self-selected velocity, the six minute walk test, falling test or the “time up and go”. These tests are simple to manage, facilitate patients collaboration by being tolerable and also reflect most of the activities that are frequently performed by elderly people. Through the use of sensors, such as accelerometers or pressure sensors, the simple walk allows not only to detect the functional mobility of elderly people, but also to quantify their spatio-temporal parameters (speed, walking time, length and width stride etc.), the joint range of motion, and to identify a small set of features for the classification of falls. Indeed, Howcroft et al. [79] proposed the use of accelerometers and plantar pressure sensors during the 7.62 m (25 feet) test to detect, from a broader set of features, a smaller one to be used for falls classification, which could further improve falls classification. Although a greater walking distance might be a more suitable indicator of everyday walking activities, the assessment of brief gait distance is commonly used in clinical practice and could allow to find a small set of features in order to improve the clinical fall risk prediction. In addition, Similä et al. [41] suggested that gait characteristics can be used to estimate the outcome of clinical assessment of dynamic stability and predict balance decline. Furthermore, walking monitored by wearable sensors could be a useful screening tool to identify people with early signs of balance deficit [103]. As shown in Figure 7, 28% of the papers includes the analysis of static condition by employing a single task, such as standing in an anatomical reference position, with open or closed eyes. These tasks allow for detecting age-related postural changes in antero-posterior or mid-lateral directions. In Turcato et al. [39], an inertial sensor has been useful to detect the angular velocity and the linear accelerations of trunk in the static condition. The results of the study highlight that it is possible to reliably predict, with a resolution by up to 400 ms, the higher frequency CoM displacements. Since the loss of static balance occurs when the CoM goes beyond the BoS, the approach described can be useful to develop wearable devices able to warn subject of the risk of falling, so that they can implement strategies to keep the balance. Furthermore, as suggested by Gopalai et al. [76], with vibrotactile feedback tools, the static evaluation of body allows to monitor and correct the postural control. In fact, they found that the use of an intelligent biofeedback system integrated by a vibrating platform is useful to improve postural control.

Additionally, as it can be seen in Table 1, two studies implement daily activities and two other studies analyze falling task. Specifically, Di Rosa et al. [72] evaluated the performance of free-activities of daily life, in order to develop a self-learning and wearable system to identify the walking decline and to detect the risks of falls at home. Furthermore, a recent paper [82] proposed a portable device able to detect falls with accuracy and to provide monitoring and timely help for the the elderly.

#### 4.4.2. Two Tasks

Of all papers included in this review, nine of them consider two tasks, as reported in Figure 7. In this case, the most adopted tests are (single and dual task) walking, sitting down and standing up. Some studies [80,81] reported in our review have explored the use of wearable sensors, the motor control and the risk of falls during the performance of a single task (walking for 7.62 m) and dual task (walking for 7.62 m with a cognitive load). These studies found that wearable sensors, such as accelerometers and pressure-sensitive insoles, can be useful, during the performance of two tasks, to detect alterations of the motor functions such as gait variability, which is indicative of greater instability and is predictive of the risk of falls. Activities like SiSt or StSi carried out daily by old people allow for detecting postural transitions, analyzing the adopted postural strategy and preventing the fall events, as reported in [75]. In [86], the authors ask to perform two tasks in order to develop a device that can assess quantitatively the degree of mobility in old people and monitor risk factors for falls.

#### 4.4.3. More than Two Tasks

In this review, seven selected papers include more than two activities. The most adopted tasks are walking, sitting down and standing up as can be seen in Figure 7. In order to improve the quality of life of elderly people, for the authors in [67], both biomechanical evaluation and detection by sensors of multiple consecutive activities are necessary, in order to reflect the static and dynamic transition period, which could predispose to a greater risk of falling if not adequately controlled. Indeed, three types of tasks (walking, standing up and falling down) were analyzed in order to monitor and to detect fall events.

Finally, five works have not been included in Figure 7 as they only provide an overview of the importance of using sensors to assess the risk of falls in the elderly, without specifying the task used for the analysis.

## 5. Conclusions

Within this manuscript, a literature review of monitoring technologies for fall risk assessment, fall prevention and fall detection in case of healthy elderly people is provided. The analysis showed that several methodologies have been proposed in the literature, and it is very difficult to find a general rule. From the perspective of sensors, most of the considered methodologies uses two sensors at maximum. Among them, accelerometers and gyroscopes are the most widespread technologies, probably because they can combine low cost and informative signals. Nevertheless, some works adopt unconventional sensors, such as radar or cameras, with the aim of investigating the effectiveness of other sensing technologies for monitoring issues. Most of them only show some preliminary results, and still require a more complete statistical validation.

Regarding the position of the sensors, the trunk is the most used segment because it plays a fundamental role both in static and dynamic stability.

By looking at the task standpoint, in case of static stability assessment, the majority of the works adopt a quiet standing test with open or closed eyes, allowing for detecting age-related postural changes in antero-posterior or mid-lateral directions. Instead, for dynamic evaluations, a walking task or a sit-to-stand test is usually adopted to detect postural variations.

Regarding the validation of sensors, the information arising from the scientific literature is reported in Table 1. However, the information about sensibility, specificity and accuracy of the considered methodologies are too diverse and do not allow for evaluating the impact of parameters such as sampling rate or sensor precision. From the analysis, there is a need for the definition of one (or more) gold standards in terms of sensors (both types and location) and tasks to be performed in order to face the extremely high variety of proposed approaches.

## Figures and Tables

**Table 1:** Summary of the wearable sensor-based systems for stability control in elderly people for the considered bibliographic research. Task types include the main activities proposed in the articles both for the dynamic as well as static analyses and reported in Table 2 and Table 3. In some cases, both methodologies have been adopted. The manuscripts have been classified according to the main identified aims, i.e., fall risk assessment (FRA), fall detection (FD) and fall prevention (FP). Acronyms for the Validation column: ACC = accuracy, Sens = sensitivity, Spec = specificity, PFA = Probability of false alarm, P_c_ = Probability of correct decision. Acronyms for the Analysis column: Dyn = Dynamic.

Author (Year)	Participants (Number/Age)	Number of Sensors	Sensor Type	Sensor Position	Task Type	Goals	Validation	Analysis
Aloqlah (2010) [63]	(3/n.a.)	1	A	HD	STN	FP, FRA	ACC ≈95%	Both
Aminian (2011) [42]	(10/26.1 ± 2.8)&(10/71 ± 4.6)	3	A, P, G	FT	SW	FP	Sens =93%, Spec =100%	Dyn
Bertolotti (2016) [64]	(18/n.a.)	4	A, P, G, M	TR, AR	SU, SD, B	FD	n.a.	Dyn
Bounyong (2016) [43]	(52/72 ± 6.1)	2	A	LG	SW	FRA	ACC =65%	Dyn
Caldara (2015) [65]	(5/31 ± 6)&(4/70.8 ± 7)	4	A, P, G, M	TR	SW	FD, FP, FRA	n.a.	Dyn
Chen (2010) [66]	(1/n.a.)	1	A	FT	SW	FP	P_c_ =86%	Dyn
Cheng (2013) [67]	(10/24 ± 2)	2	A, EMG	LG	SW, SU, SD	FD	Sens = 95.33%, Spec = 97.66%	Dyn
Cola (2015) [68]	(30/32.9 ± 12.2)	1	A	TR	SW	FD, FRA	ACC = 84%	Dyn
Crispim-Junior (2013) [69]	(29/65)	1	C	EXT	SW, DA	FD	Sens = 88.33%	Dyn
Curone (2010) [70]	(6/29.5)	1	A	TR	SU, SD, SW	FD	P_c_ ≥90%	Both
De la Guia Solaz (2010) [71]	(10/23.7 ± 2.2)&(10/77.2 ± 4.3)	2	A, P	TR	SU, SD, SW, F	FD	ACC =100%, P_c_=93%, PFA=29%	Dyn
Deshmukh (2012) [40]	(4/n.a.)	3	A, G, M	LG	STN	FRA	n.a.	Static
Di Rosa (2017) [72]	(29/71.1 ± 6.9)	2	A, P	FT	DA	FRA	ACC =95%	Dyn
Diraco (2014) [73]	(18/38 ± 6)	1	T	EXT	STN	FD	P_c_ >83%	Static
Fernandez-Luque (2010) [74]	(n.a./n.a.)	4	A, P, M, IR	EXT	DA	FD, FRA	n.a.	Dyn
Ganea (2012) [75]	(35/54.2 ± 5.7)	2	A, G	TR, LG	SU, SD	FD, FP, FRA	ACC = 95%	Dyn
Gopalai (2011) [76]	(12/23.45 ± 1.45)	2	A, G	TR	STN	FP, FRA	n.a.	n.a.
Greene (2011) [77]	(114/71 ± 6.6)	2	A, G	LG	SW	FD	n.a.	Dyn
Hegde (2015) [78]	(n.a./n.a.)	3	A, P, G	FT	n.a.	FD, FRA	n.a.	Dyn
Howcroft (2017) [79]	(100/75.5 ± 6.7)	2	A, P	TR, HD, LG, FT	SW	FP, FRA	ACC =78%, Sens =26%, Spec =95%	Dyn
Howcroft (2017) [80]	(76/75.2 ± 6.6)	2	A, P	TR, HD, LG, FT	SW, DW	FP, FRA	ACC =57%, Sens =43%, Spec =65%	Dyn
Howcroft (2016) [81]	(100/75.5 ± 6.7)	2	A, P	TR, HD, LG, FT	SW, DW	FD, FP, FRA	n.a.	Dyn
Jian (2015) [82]	(8/33)	2	A, G	TR	F	FD	n.a.	Dyn
Jiang (2011) [83]	(48/40)	3	A, P, C	n.a.	SW, STN	FP, FRA	n.a.	Dyn
Karel (2010) [84]	(41/24 ± 4)&(50/67 ± 5)	1	A	TR	SW	FD	Sens =98.4%, Spec =99.9%	Dyn
Micó-Amigo (2016) [85]	(20/73.7 ± 7.9)	2	A, G	TR, LG	SW	FD, FP, FRA	Sens =92.6÷98.2%	Dyn
Najafi (2002) [86]	(11/79 ± 6)	1	G	TR	SU, SD	FRA	Sens ≥95%, Spec ≥95%	Dyn
Ozcan (2016) [87]	(n.a./n.a.)	2	A, G	TR	n.a.	FD	Sens =96.36%, Spec =92.45%	Static
Paoli (2011) [88]	(1/n.a.)	>4	A, P, M, IR	TR	DA	FD	n.a.	Both
Qu (2016) [89]	(10/25)	1	A	TR	F	FD	ROC curve	Dyn
Sazonov (2013) [90]	(1/n.a.)	2	A, P	FT	STN, STT, SW	FD, FRA	n.a.	Both
Simila (2017) [41]	(42/74.17 ± 5.57)	1	A	TR	SW	FP, FRA	Sens =80%, Spec =73%	Dyn
Stone (2013) [91]	(15/67)	1	K	n.a.	SW	FD	n.a.	Dyn
Szurley (2009) [92]	(n.a./n.a.)	1	A	TR	n.a.	FP	n.a.	Dyn
Tamura (2005) [93]	(6/66.3 ± 5)	1	A	TR	SU, SD	FD	P_c_ =86%	Dyn
Tang (2016) [94]	(1/n.a.)	1	R	LG	SW, STR	FD, FP	n.a.	Dyn
Turcato (2010) [39]	(5/26 ± 6)	2	A, W	TR	STN	FP	ACC =55−70%	Static
Van de Ven (2015) [95]	(1 /n.a.)	2	A, P	FT	STN, STT	FD	n.a.	Dyn
van Schooten (2016) [96]	(319/75.5 ± 6.9)	1	A	TR	DA	FD, FP, FRA	n.a.	Dyn
Vincenzo (2016) [97]	(57/74.35 ± 6.53)	1	A	TR	STN	FD	n.a.	Static
Yao (2015) [98]	(9/25)	3	A, G, M	TR	SW, F, R	FD, FP, FRA	n.a.	Dyn
Yuan (2015) [99]	(n.a./n.a.)	2	A, G	TR	F, STT, L	FD	n.a.	Both

**Table 2:** Legend of acronyms for the tasks in Table 1.

Task Type	Acronym
Standing	STN
Single Task Walking	SW
Dual Task Walking	DW
Standing Up	SU
Sitting Down	SD
Bending	B
Daily Activities	DA
Falling	F
Running	R
Sitting	STT
Lying	L
Stairs	STR

**Table 3:** Legend of acronyms for the sensor positions in Table 1.

Sensor Position	Anatomical Location	Acronym
Head		HD
Foot	Shoes, heel	FT
Trunk	L3, L5, sternum, waist, pelvis, neck, chest	TR
Arm	Wrist, forearm	AR
Leg	Thigh, cruris, ankle, shank, knee	LG
External		EXT

**Table 4:** Legend of acronyms for the sensors types in Table 1.

Sensor Type	Acronym
Accelerometer	A
Gyroscope	G
Pressure sensors	P
Magnetometer	M
Radar	R
Time-of-flight (TOF) Camera	T
Kinect console	K
Wii console	W
Electromyography	EMG
Infrared sensors	IR

## Abbreviations

The following abbreviations are used in this manuscript:

BoS: Base of Support

CoG: Center of Gravity

CoM: Center of Mass

CoP: Center of Pressure
