# Correction: Rucco, R.; et al. Type and Location of Wearable Sensors for Monitoring Falls during Static and Dynamic Tasks in Healthy Elderly: A Review. *Sensors* 2018, *18*, 1613

**DOI:** 10.3390/s18082462

**Published:** 2018-07-30

**Authors:** Rosaria Rucco, Antonietta Sorriso, Marianna Liparoti, Giampaolo Ferraioli, Pierpaolo Sorrentino, Michele Ambrosanio, Fabio Baselice

**Affiliations:** 1 Department of Motor Sciences and Wellness, University of Naples “Parthenope”, 80133 Naples, Italy; marianna.liparoti@uniparthenope.it; 2 IDC Hermitage Capodimonte, 80133 Naples, Italy; pierpaolo.sorrentino@uniparthenope.it; 3 Department of Engineering, University of Naples “Parthenope”, 80133 Naples, Italy; antonietta.sorriso@uniparthenope.it (A.S.); michele.ambrosanio@uniparthenope.it (M.A.); fabio.baselice@uniparthenope.it (F.B.); 4 Department of Science and Technologies, University of Naples “Parthenope”, 80133 Naples, Italy; giampaolo.ferraioli@uniparthenope.it

The authors wish to make a correction to their paper [1]. The following Table 1 should be replaced with the table shown below it.

The authors would like to apologize for any inconvenience caused to the readers by these changes. The changes do not affect the scientific results. The manuscript will be updated and the original will remain online on the article webpage, with a reference to this Correction.

## Figures and Tables

**Table 1:** Summary of the wearable sensor-based systems for stability control in elderly people for the considered bibliographic research. Task types include the main activities proposed in the articles both for the dynamic as well as static analyses and reported in Tables 2 and 3. In some cases, both methodologies have been adopted. The manuscripts have been classified according to the main identified aims, i.e. fall risk assessment (FRA), fall detection (FD) and fall prevention (FP). Acronyms for the Validation column: ACC = accuracy, Sens = sensitivity, Spec = specificity, PFA = Probability of false alarm, P_c_ = Probability of correct decision. Acronyms for the Analysis column: Dyn = Dynamic.

Author (Year)	Participants (Number/Age)	Number of Sensors	Sensor Type	Sensor Position	Task Type	Goals	Validation	Analysis
Aloqlah (2010) [63]	(3/n.a.)	1	A	HD	STN	FP, FRA	ACC ≈ 95%	Both
Aminian (2011) [42]	(10/26.1 ± 2.8)&(10/71 ± 4.6)	3	A, P, G	FT	SW	FP	Sens = 93%, Spec = 100%	Dyn
Bertolotti (2016) [64]	(18/n.a.)	4	A, P, G, M	TR, AR	SU, SD, B	FD	n.a.	Dyn
Bounyong (2016) [43]	(52/72 ± 6.1)	2	A	LG	SW	FRA	ACC = 65%	Dyn
Caldara (2015) [65]	(5/31 ± 6)&(4/70.8 ± 7)	4	A, P, G, M	TR	SW	FD, FP, FRA	n.a.	Dyn
Chen (2010) [66]	(1/n.a.)	1	A	FT	SW	FP	P_c_ = 86%	Dyn
Cheng (2013) [67]	(10/24 ± 2)	2	A, EMG	LG	SW, SU, SD	FD	Sens = 95.33%, Spec = 97.66%	Dyn
Cola (2015) [68]	(30/32.9 ± 12.2)	1	A	TR	SW	FD, FRA	ACC = 84%	Dyn
Crispim-Junior (2013) [69]	(29/65)	1	C	EXT	SW, DA	FD	Sens = 88.33%	Dyn
Curone (2010) [70]	(6/29.5)	1	A	TR	SU, SD, SW	FD	P_c_ ≥ 90%	Both
De la Guia Solaz (2010) [71]	(10/23.7 ± 2.2)&(10/77.2 ± 4.3)	2	A, P	TR	SU, SD, SW, F	FD	ACC = 100%, P_c_ = 93%, *PFA* = 29%	Dyn
Deshmukh (2012) [40]	(4/n.a.)	3	A, G, M	LG	STN	FRA	n.a.	Static
Di Rosa (2017) [72]	(29/71.1 ± 6.9)	2	A, P	FT	DA	FRA	ACC = 95%	Dyn
Diraco (2014) [73]	(18/38 ± 6)	1	T	EXT	STN	FD	P_c_ > 83%	Static
Fernandez-Luque (2010) [74]	(n.a./n.a.)	4	A, P, M, IR	EXT	DA	FD, FRA	n.a.	Dyn
Ganea (2012) [75]	(35/54.2 ± 5.7)	2	A, G	TR, LG	SU, SD	FD, FP, FRA	ACC = 95%	Dyn
Gopalai (2011) [76]	(12/23.45 ± 1.45)	2	A, G	TR	STN	FP, FRA	n.a.	n.a.
Greene (2011) [77]	(114/71 ± 6.6)	2	A, G	LG	SW	FD	n.a.	Dyn
Hegde (2015) [78]	(n.a./n.a.)	3	A, P, G	FT	n.a.	FD, FRA	n.a.	Dyn
Howcroft (2017) [79]	(100/75.5 ± 6.7)	2	A, P	TR, HD, LG, FT	SW	FP, FRA	ACC = 78%, Sens = 26%, Spec = 95%	Dyn
Howcroft (2017) [80]	(76/75.2 ± 6.6)	2	A, P	TR, HD, LG, FT	SW, DW	FP, FRA	ACC = 57%, Sens = 43%, Spec = 65%	Dyn
Howcroft (2016) [81]	(100/75.5 ± 6.7)	2	A, P	TR, HD, LG, FT	SW, DW	FD, FP, FRA	n.a.	Dyn
Jian (2015) [82]	(8/33)	2	A, G	TR	F	FD	n.a.	Dyn
Jiang (2011) [83]	(48/40)	3	A, P, C	n.a.	SW, STN	FP, FRA	n.a.	Dyn
Karel (2010) [84]	(41/24 ± 4)&(50/67 ± 5)	1	A	TR	SW	FD	Sens = 98.4%, Spec = 99.9%	Dyn
Micó-Amigo (2016) [85]	(20/73.7 ± 7.9)	2	A, G	TR, LG	SW	FD, FP, FRA	Sens = 92.6 ÷ 98.2%	Dyn
Najafi (2002) [86]	(11/79 ± 6)	1	G	TR	SU, SD	FRA	Sens ≥ 95%, Spec ≥ 95%	Dyn
Ozcan (2016) [87]	(n.a./n.a.)	2	A, G	TR	n.a.	FD	Sens = 6.36%, Spec = 92.45%	Static
Paoli (2011) [88]	(1/n.a.)	>4	A, P, M, IR	TR	DA	FD	n.a.	Both
Qu (2016) [89]	(10/25)	1	A	TR	F	FD	ROC curve	Dyn
Sazonov (2013) [90]	(1/n.a.)	2	A, P	FT	STN, STT, SW	FD, FRA	n.a.	Both
Simila (2017) [41]	(42/74.17 ± 5.57)	1	A	TR	SW	FP, FRA	Sens = 80%, Spec = 73%	Dyn
Stone (2013) [91]	(15/67)	1	K	n.a.	SW	FD	n.a.	Dyn
Szurley (2009) [92]	(n.a./n.a.)	1	A	TR	n.a.	FP	n.a.	Dyn
Tamura (2005) [93]	(6/66.3 ± 5)	1	A	TR	SU, SD	FD	P_c_ = 86%	Dyn
Tang (2016) [94]	(1/n.a.)	1	R	LG	SW, STR	FD, FP	n.a.	Dyn
Turcato (2010) [39]	(5/26 ± 6)	2	A, W	TR	STN	FP	ACC = 55–70%	Static
Van de Ven (2015) [95]	(1 /n.a.)	2	A, P	FT	STN, STT	FD	n.a.	Dyn
van Schooten (2016) [96]	(319/75.5 ± 6.9)	1	A	TR	DA	FD, FP, FRA	n.a.	Dyn
Vincenzo (2016) [97]	(57/74.35 ± 6.53)	1	A	TR	STN	FD	n.a.	Static
Yao (2015) [98]	(9/25)	3	A, G, M	TR	SW, F, R	FD, FP, FRA	n.a.	Dyn
Yuan (2015) [99]	(n.a./n.a.)	2	A, G	TR	F, STT, L	FD	n.a.	Both

**Table 1:** Summary of the wearable sensor-based systems for stability control in elderly people for the considered bibliographic research. Task types include the main activities proposed in the articles both for the dynamic as well as static analyses and reported in Tables 2 and 3. In some cases, both methodologies have been adopted. The manuscripts have been classified according to the main identified aims, i.e. fall risk assessment (FRA), fall detection (FD) and fall prevention (FP). Acronyms for the Validation column: ACC = accuracy, Sens = sensitivity, Spec = specificity, PFA = Probability of false alarm, P_c_ = Probability of correct decision. Acronyms for the Analysis column: Dyn = Dynamic.

Author (Year)	Participants (Number/Age)	Number of Sensors	Sensor Type	Sensor Position	Task Type	Goals	Validation	Analysis
Aloqlah (2010) [63]	(3/n.a.)	1	A	HD	STN	FP, FRA	ACC ≈ 95%	Both
Aminian (2011) [42]	(10/26.1 ± 2.8)&(10/71 ± 4.6)	3	A, P, G	FT	SW	FP	Sens = 93%, Spec = 100%	Dyn
Bertolotti (2016) [64]	(18/n.a.)	4	A, P, G, M	TR, AR	SU, SD, B	FD	n.a.	Dyn
Bounyong (2016) [43]	(52/72 ± 6.1)	2	A	LG	SW	FRA	ACC = 65%	Dyn
Caldara (2015) [65]	(5/31 ± 6)&(4/70.8 ± 7)	4	A, P, G, M	TR	SW	FD, FP, FRA	n.a.	Dyn
Chen (2010) [66]	(1/n.a.)	1	A	FT	SW	FP	P_c_ = 86%	Dyn
Cheng (2013) [67]	(10/24 ± 2)	2	A, EMG	LG	SW, SU, SD	FD	Sens = 95.33%, Spec = 97.66%	Dyn
Cola (2015) [68]	(30/32.9 ± 12.2)	1	A	TR	SW	FD, FRA	ACC = 84%	Dyn
Crispim-Junior (2013) [69]	(29/65)	1	C	EXT	SW, DA	FD	Sens = 88.33%	Dyn
Curone (2010) [70]	(6/29.5)	1	A	TR	SU, SD, SW	FD	P_c_ ≥ 90%	Both
De la Guia Solaz (2010) [71]	(10/23.7 ± 2.2)&(10/77.2 ± 4.3)	2	A, P	TR	SU, SD, SW, F	FD	ACC 100%, P_c_ = 93%, *PFA* = 29%	Dyn
Deshmukh (2012) [40]	(4/n.a.)	3	A, G, M	LG	STN	FRA	n.a.	Static
Di Rosa (2017) [72]	(29/71.1 ± 6.9)	2	A, P	FT	DA	FRA	ACC = 95%	Dyn
Diraco (2014) [73]	(18/38 ± 6)	1	T	EXT	STN	FD	P_c_ > 83%	Static
Fernandez-Luque (2010) [74]	(n.a./n.a.)	4	A, P, M, IR	EXT	DA	FD, FRA	n.a.	Dyn
Ganea (2012) [75]	(35/54.2 ± 5.7)	2	A, G	TR, LG	SU, SD	FD, FP, FRA	ACC = 95%	Dyn
Gopalai (2011) [76]	(12/23.45 ± 1.45)	2	A, G	TR	STN	FP, FRA	n.a.	n.a.
Greene (2011) [77]	(114/71 ± 6.6)	2	A, G	LG	SW	FD	n.a.	Dyn
Hegde (2015) [78]	(n.a./n.a.)	3	A, P, G	FT	n.a.	FD, FRA	n.a.	Dyn
Howcroft (2017) [79]	(100/75.5 ± 6.7)	2	A, P	TR, HD, LG, FT	SW	FP, FRA	ACC = 78%, Sens = 26%, Spec = 95%	Dyn
Howcroft (2017) [80]	(76/75.2 ± 6.6)	2	A, P	TR, HD, LG, FT	SW, DW	FP, FRA	ACC = 57%, Sens = 43%, Spec = 65%	Dyn
Howcroft (2016) [81]	(100/75.5 ± 6.7)	2	A, P	TR, HD, LG, FT	SW, DW	FD, FP, FRA	n.a.	Dyn
Jian (2015) [82]	(8/33)	2	A, G	TR	F	FD	n.a.	Dyn
Jiang (2011) [83]	(48/40)	3	A, P, C	n.a.	SW, STN	FP, FRA	n.a.	Dyn
Karel (2010) [84]	(41/24 ± 4)&(50/67 ± 5)	1	A	TR	SW	FD	Sens = 98.4%, Spec =99.9%	Dyn
Micó-Amigo (2016) [85]	(20/73.7 ± 7.9)	2	A, G	TR, LG	SW	FD, FP, FRA	n.a.	Dyn
Najafi (2002) [86]	(11/79 ± 6)	1	G	TR	SU, SD	FRA	Sens ≥ 95%, Spec ≥ 95%	Dyn
Ozcan (2016) [87]	(n.a./n.a.)	2	A, G	TR	n.a.	FD	Sens = 96.36%, Spec = 92.45%	Static
Paoli (2011) [88]	(1/n.a.)	>4	A, P, M, IR	TR	DA	FD	n.a.	Both
Qu (2016) [89]	(10/25)	1	A	TR	F	FD	ROC curve	Dyn
Sazonov (2013) [90]	(1/n.a.)	2	A, P	FT	STN, STT, SW	FD, FRA	n.a.	Both
Simila (2017) [41]	(42/74.17 ± 5.57)	1	A	TR	SW	FP, FRA	Sens = 80%, Spec = 73%	Dyn
Stone (2013) [91]	(15/67)	1	K	n.a.	SW	FD	n.a.	Dyn
Szurley (2009) [92]	(n.a./n.a.)	1	A	TR	n.a.	FP	n.a.	Dyn
Tamura (2005) [93]	(6/66.3 ± 5)	1	A	TR	SU, SD	FD	P_c_ = 86%	Dyn
Tang (2016) [94]	(1/n.a.)	1	R	LG	SW, STR	FD, FP	n.a.	Dyn
Turcato (2010) [39]	(5/26 ± 6)	2	A, W	TR	STN	FP	ACC = 55–70%	Static
Van de Ven (2015) [95]	(1 /n.a.)	2	A, P	FT	STN, STT	FD	n.a.	Dyn
van Schooten (2016) [96]	(319/75.5 ± 6.9)	1	A	TR	DA	FD, FP, FRA	n.a.	Dyn
Vincenzo (2016) [97]	(57/74.35 ± 6.53)	1	A	TR	STN	FD	n.a.	Static
Yao (2015) [98]	(9/25)	3	A, G, M	TR	SW, F, R	FD, FP, FRA	n.a.	Dyn
Yuan (2015) [99]	(n.a./n.a.)	2	A, G	TR	F, STT, L	FD	n.a.	Both

## References

[1] Rucco R., Sorriso A., Liparoti M., Ferraioli G., Sorrentino P., Ambrosanio M., Baselice F. (2018). Type and Location of Wearable Sensors for Monitoring Falls during Static and Dynamic Tasks in Healthy Elderly: A Review. Sensors.

